# Adult brain abscess associated with patent foramen ovale: a case report

**DOI:** 10.1186/1752-1947-1-68

**Published:** 2007-08-24

**Authors:** Georgios T Stathopoulos, Christina G Mandila, Georgios V Koukoulitsios, Nikodimos G Katsarelis, Michel Pedonomos, Andreas Karabinis

**Affiliations:** 1 Intensive Care Unit, General Hospital "G. Gennimatas", Athens, Greece

## Abstract

Brain abscess results from local or metastatic septic spread to the brain. The primary infectious site is often undetected, more commonly so when it is distant. Unlike pediatric congenital heart disease, minor intracardiac right-to-left shunting due to patent foramen ovale has not been appreciated as a cause of brain abscess in adults. Here we present a case of brain abscess associated with a patent foramen ovale in a 53-year old man with dental-gingival sepsis treated in the intensive care unit. Based on this case and the relevant literature we suggest a link between a silent patent foramen ovale, paradoxic pathogen dissemination to the brain, and development of brain abscess.

## Background

One of the functions of the lung vasculature is to mechanically filter the blood; hence right-to-left circulatory shunts can serve as entrance gates for bland or septic thrombi into the arterial circulation [1-3]. Pediatric brain abscess secondary to paradoxic infection via congenital intracardiac shunts, as well as adult brain abscess resulting from extracardiac shunts, such as pulmonary arteriovenous malformation (PAVM), are well recognized [4-6]. On the contrary, clinically silent intracardiac shunting due to patent foramen ovale (PFO) has not been appreciated as a source of septic brain emboli in adults. However, autopsy studies have detected a PFO in 20–35% of adults, and a cross-sectional study has implicated PFOs in the pathogenesis of embolic stroke [7-9]. Hence a PFO may be sufficient for the development of brain abscess in clinical circumstances when bacteremia of the venous circulation occurs [10].

## Case presentation

A 53-yr-old farmer presented 8 days after having suffered a seizure. He reported another seizure 2 months before, for which he did not seek medical attention. His past history included morbid obesity (body mass index: 51.5), a right bundle branch block, heavy nicotine addiction (65 pack-years; Fagerstrom test score = 8), chronic obstructive pulmonary disease (COPD), and trigeminal neuralgia for the last ten years. His dental health was impaired with dental, periodontal, and gingival sepsis and a recent history of dental procedures, for which he did not take antibiotics.

At presentation temperature was 37.7°C, heart rate was 88/min, respiratory rate was 15/min, and blood pressure was 123/72 mmHg. The patient showed no abnormal physical signs except from purulent gingival drainage and several septic teeth, and was alert and oriented with a Glascow Coma Scale score of 15/15. Pupil sizes, light reflexes, extra-ocular movements, and visual fields were normal. Cranial nerves and motor-sensor functions were unimpaired. Tendon reflexes were intact and plantar responses were flexor.

**Figure 1:**
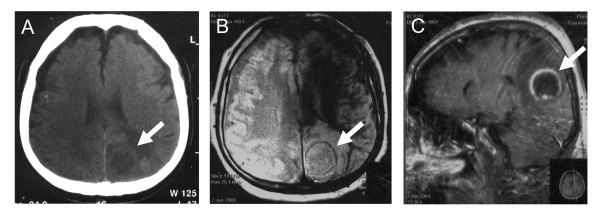
A 53-year-old man with reported seizures was evaluated. (A) Head CT without contrast medium reveals a round left occipital mass lesion (arrow) with hyperdense margins and a hypodense center. (B) A T-2-weighted head MRI image without contrast shows a mass (arrow) with high central signal intensity, a ring of heterogeneous peripheral signal intensity similar to that of the brain parenchyma, and a surrounding area of bright signal in the white-matter tracts. (C) On the contrast-enhanced T-1-weighted head MRI image, the mass has low signal intensity centrally that suggests the presence of fluid, and is surrounded by an enhancement ring, beyond which extends an area of low signal that indicates edema.

The patient's urine examination, peripheral blood cell counts, serum biochemistry, and clotting times were unremarkable. Serum C-reactive protein (85 U/l) and procalcitonin (8 U/l) levels were elevated. Electrocardiography showed normal rhythm, a rate of 85/min, the known heart block, and no changes from a previous test. Arterial blood gases and a chest X-ray were normal (not shown). Electroencephalography was inconclusive. A computed tomography (CT)-scan of the brain without intravenous contrast medium due to a history of allergy, depicted a left occipital mass lesion measuring 25 × 30 mm, featuring a more radiolucent center with a denser periphery (Figure 1A). Brain magnetic resonance imaging (MRI) showed a mass lesion on T2-weighted images, with a periphery exhibiting a higher signal on contrast-enhanced T1-weighted images (Figures 1B, 1C).

**Figure 2:**
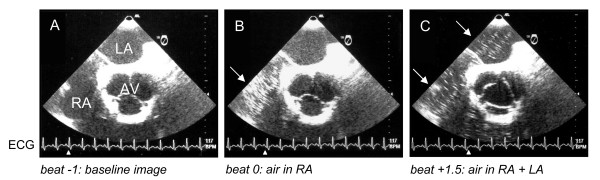
The presence of abnormal circulatory shunting was determined by transesophageal bubble-contrast echocardiography. The timing of image acquisition is indicated by the white arrowheads on the ECG strip and explained by the text below the images. (A) Posterior transverse view before agitated saline-air mixture injection (RA = right atrium; LA = left atrium; AV = aortic valve). (B) During agitated saline-air mixture injection bubbles are visible only in the right atrium (white arrow). (C) One and a half heart-beat after agitated saline-air mixture injection air bubbles are detected both in the right and left atria (white arrows).

Brain abscess was ruled in, and the patient was placed on phenytoin, antibiotics, and admitted to the intensive care unit (ICU). Trans-esophageal bubble-contrast echocardiography revealed shunting of air bubbles injected via a subclavian vein catheter directly from the right atrium to the left atrium and ventricle within 1 heart-beat after injection, confirming the existence of a PFO (Figure 2).

Subsequently, surgical removal of the brain abscess was performed. Gram staining of the abscess' content revealed multiple gram positive cocci forming clusters, and Staphylococcus haemolyticus was cultured. The same microorganism was isolated from the patient's gingival smears and blood. Meropenem 2 gr i.v. t.i.d., metronidazole 500 mg i.v. q.i.d., and teicoplanin 1 gr i.v. b.i.d. were initiated empirically, and continued based on in vitro sensitivity testing. The patient improved very slowly, and was discharged on the 120^th ^day of hospital stay. After two years of follow-up he has recovered almost completely, still showing residual lower limb weakness.

## Discussion

The pathogenesis of brain abscess is largely comprehended in terms of the route of pathogen dissemination to the brain. One route is local; trauma or infection of adjacent structures (eg venous sinuses of the brain, paranasal sinuses) can lead to brain infection by direct invasion of contiguous anatomical layers [10]. An additional route is systemic; infected thrombi in the systemic arterial circulation (e.g. left-sided endocarditis) or having direct access to the systemic circulation (eg PAVMs) directly embolize the brain [6,10]. However, a fraction of brain abscesses are poorly explained by the above, occuring in patients with infectious sites lacking direct access to the systemic circulation (eg right endocarditis, septic thrombophlebitis, odontogenic or intraabdominal abscess). A PFO may precipitate the development of brain abscess in these cases [11].

The foramen ovale serves as a physiologic right-to-left intracardiac shunt during intrauterine life. Early after birth it is occluded and remains shut by a left-to-right atrial pressure gradient in healthy individuals; anatomic closure is thought to occur with time [1]. Older studies have detected a patent foramen ovale in a small fraction of healthy individuals [7,8]. Moreover, a functionally shut foramen ovale may open in healthy individuals during a Valsava maneuver or in disease states that cause pulmonary hypertension (e.g. COPD) [8,9]. A clinically silent PFO has been associated with embolic stroke and has been proposed as a precipitating cause of brain abscess in otherwise healthy adults [3,9,10]. However, this concept has not been proven and the number of reported cases is low.

A PubMed search using the keywords "patent foramen ovale" AND "brain abscess" yielded 26 articles (20 Jul 2007), out of which nine described ten patients with brain abscess not explained otherwise than on a basis of a silent PFO [3,10,11]. Out of these ten patients, only one had obvious perianal sepsis, while the rest had no prominent septic source [12]. Symptoms ranged from none to focal cranial nerve deficit to lethargy [10-13]. Trans-esophageal echocardiography was the diagnostic test of choice, revealing early-appearing air bubbles in the left atrium, and, in most cases, no additional findings, except from one case of coexisting atrial vegetations [14].

## Conclusion

Here we report the case of a patient with dental-gingival sepsis that developed a brain abscess in the community. This patient had no precipitating factors for brain abscess, other than a PFO detected by bubble-contrast echocardiography. The association of the brain abscess with dental sepsis was suggested by isolation of the same bacterial strain from the abscess, blood, and gingival pus. The factors that led to opening of the foramen ovale are not known; however, increased pulmonary arterial pressure due to COPD can provide a mechanism [8,9]. This and other reports collectively suggest that PFO may be implicated in the pathogenesis of brain abscess, pending larger case-control studies that would be required for proof-of-concept. If this link were true, clinically silent PFO may pose serious risks to otherwise healthy, non-immunocompromised individuals, by precipitating embolic stroke and brain abscess.

## Abbreviations

PAVM, pulmonary arteriovenous malformation

PFO, patent foramen ovale

COPD, chronic obstructive pulmonary disease

CT, computed tomography

MRI, magnetic resonance imaging

ICU, intensive care unit

## Competing interests

The author(s) declare that they have no competing interests.

## Authors' contributions

GTS wrote the manuscript. CGM, GVK, NGK, MP, and AK collected the data and helped to draft the manuscript. All authors read and approved the final manuscript.
